# P-1236. Vancomycin Population Pharmacokinetics Using Volumetric Absorptive Microsampling in Critically Ill Children with Multiple Organ Dysfunction Syndrome

**DOI:** 10.1093/ofid/ofae631.1418

**Published:** 2025-01-29

**Authors:** Justin Shiau, Victor Amajor, Nathaniel J Rhodes, Athena Zuppa, Kevin J Downes, John Takyi-Williams, Bo Wen, Marc H Scheetz

**Affiliations:** Midwestern University Pharmacometrics Center of Excellence, Downers Grove, Illinois; Children's Hospital of Philadelphia, Philadelphia, Pennsylvania; Midwestern University, Downers Grove, IL; Children's Hospital of Phildaelphia, Medford, New Jersey; Children's Hospital of Philadelphia, Philadelphia, Pennsylvania; TSRL Inc, Ann Arbor, Michigan; University of Michigan, Ann Arbor, Michigan; Midwestern University, Downers Grove, IL

## Abstract

**Background:**

Vancomycin (VAN) is the most frequently used antibiotic for hospitalized patients in the United States. Wide use occurs as it is a first line treatment for severe gram-positive bacterial infections in both adults and pediatrics. Most VAN dosing in children with multiple organ dysfunction syndrome (MODS) is guided via population pharmacokinetic (PK) approaches based on creatinine clearance equations and frequent samplings. Volumetric absorptive microsampling (VAMS) offers sampling procedures that require as little as 20 mcL of blood. The primary objective of this study was to develop a population PK (popPK) model for VAN in children with MODS using VAMS.

**Table 1.**
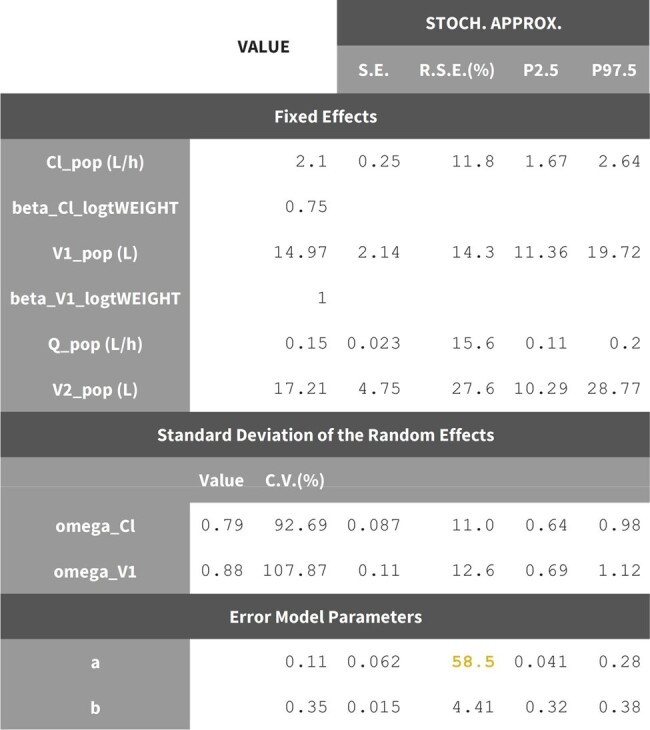
Population parameter estimation values.

**Methods:**

We conducted a multi-center prospective observational study of VAN PK in pediatric MODS. Subjects (< 18 years) participating in a larger observational study of children with MODS in the ICU were eligible. Up to 15 PK samples were collected over 3 consecutive days, along with pertinent covariate data. Parametric popPK modeling was performed using Monolix 2024R1. Covariate selection was based on improvements in objective function value and physiologic relevance. Children on CRRT were excluded from the present analyses. Subjects in our interim analysis were also excluded from analysis if they were missing demographic information or were pending data queries on VAN concentrations.

**Figure 1.**
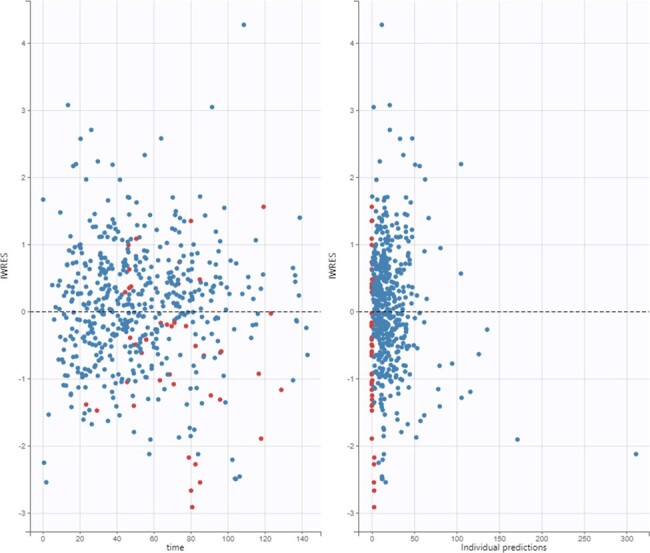
Individual weighted residuals according to time and individual predictions.

**Results:**

52 subjects from 8 sites were included with a median age of 6.5 y (range: 2 m–17 y) and a median weight of 24.5 kg (range: 3-214). A two-compartment model with clearance adjusted for allometric scaling and estimated glomerular function best described the data. Population predictions were modest (R2=0.28) whereas individualized predictions were improved (R2=0.61). Between subject variation for clearance and volume of distribution was high even after correcting for U25 creatinine clearance (92.7% and 107.9%, CV% respectively).

**Figure 2.**
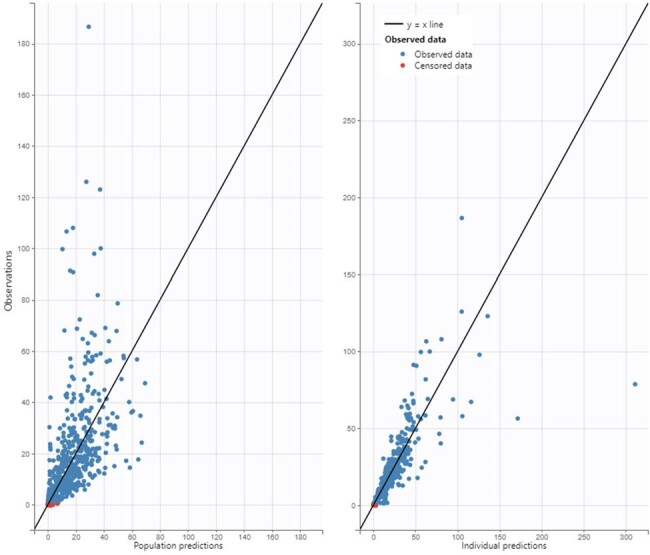
Observed vs. population (A) and individual (B) predictions for the model.

**Conclusion:**

Children with MODS display high between subject variability of VAN clearance and volume of distribution. Frequent sampling is needed because “stead-state” does not exist in the early period of MODS for children. VAMS can facilitate small volume sampling and future Bayesian models based on data such as these can facilitate fewer sampling events to classify the patient’s dynamic clearance.

**Disclosures:**

**Nathaniel J. Rhodes, PharmD MS**, Apothecademy, LLC: Advisor/Consultant **Kevin J. Downes, MD**, Paratek, Inc.: Grant/Research Support|Veloxis Pharmaceuticals, Inc.: Grant/Research Support **Marc H. Scheetz, PharmD, MSc**, Abbvie: Advisor/Consultant|Basilea: Advisor/Consultant|Cidara: Advisor/Consultant|DoseMe: Advisor/Consultant|Entasis: Advisor/Consultant|F2G: Advisor/Consultant|GSK: Advisor/Consultant|Lykos: Advisor/Consultant|Roche: Advisor/Consultant|Third Pole Therapeutics: Advisor/Consultant|Xelia: Advisor/Consultant

